# *Lactiplantibacillus plantarum* 124 Modulates Sleep Deprivation-Associated Markers of Intestinal Barrier Dysfunction in Mice in Conjunction with the Regulation of Gut Microbiota

**DOI:** 10.3390/nu15184002

**Published:** 2023-09-15

**Authors:** Longyan Li, Lei Wu, Tong Jiang, Tingting Liang, Lingshuang Yang, Ying Li, He Gao, Jumei Zhang, Xinqiang Xie, Qingping Wu

**Affiliations:** 1 College of Food Science, South China Agricultural University, Guangzhou 510642, China; 18868006204@163.com (L.L.); jt0925@stu.scau.edu.cn (T.J.); yangls8272@163.com (L.Y.); 2 Guangdong Provincial Key Laboratory of Microbial Safety and Health, State Key Laboratory of Applied Microbiology Southern China, Key Laboratory of Agricultural Microbiomics and Precision Application, Ministry of Agriculture and Rural Affairs, Institute of Microbiology, Guangdong Academy of Sciences, Guangzhou 510070, China; wuleigdim@163.com (L.W.); gdim_liangtt@outlook.com (T.L.); liying@gdim.cn (Y.L.); gaohe.881128@163.com (H.G.); zhangjm926@126.com (J.Z.)

**Keywords:** sleep disorder, fecal microbiota transplantation, intestinal, gut microbiome, oxidative stress, inflammation

## Abstract

Intestinal diseases caused by sleep deprivation (SD) are severe public health threats worldwide. However, whether or not probiotics attenuate the intestinal damage associated with SD remains unclear. In this study, we used antibiotic pretreatment and fecal microbiota transplantation to investigate the protective role of *Lactiplantibacillus plantarum* (*L. plantarum*) 124 against SD-related intestinal barrier damage in C57BL/6 mice. Compared with those of a normal sleeping mouse, we observed that intestinal antioxidant capacity and anti-inflammatory cytokine levels were decreased, while pro-inflammatory cytokines were increased in sleep deprivation mice with an increasing duration of sleep deprivation. This resulted in decreased tight junction protein expression and increased intestinal barrier permeability. In contrast, intragastric administration with *L. plantarum* 124 reversed SD-associated intestinal oxidative stress, inflammation, colonic barrier damage, and the dysbiosis of the microbiota in the colon. In addition, *L. plantarum* 124 restored gut microbiota homeostasis via restoring abundance, including that of *Dubosiella*, *Faecalibaculum*, *Bacillus*, *Lachnoclostridium*, and *Bifidobacterium*. Further studies showed that gut microbiota mediated SD-associated intestinal damage and the treatment *L. plantarum* 124 in SD-associated colonic barrier damage. *L. plantarum* 124 is a potential candidate for alleviating SD-associated intestinal barrier damage. Overall, *L. plantarum* 124 consumption attenuates intestinal oxidative stress, inflammation, and intestinal barrier damage in SD-associated mice via the modulation of gut microbes.

## 1. Introduction

Sleep is the most fundamental physiological activity. Healthy sleep is critical to brain development, immune function, cognitive function, and metabolism. Studies have shown that the worldwide prevalence of sleep disturbance during the novel COVID-19 pandemic was approximately 35 ± 5% [1,2,3], with the incidence of sleep deprivation being as high as (52–75%) in patients with COVID-19 infection [1,2,3]. Clinical and experimental studies have reported that SD was linked to a wide range of conditions such as cardiovascular disease, depression, diabetes, obesity, high blood pressure, hyperlipidemia, and Alzheimer’s disease [4,5]. In addition, sleep deprivation has also been associated with digestive disorders. Recent studies found that sleep deprivation causes the accumulation of reactive oxygen species (ROS) in the gut, leading to many inflammatory bowel diseases and even premature death [6,7,8]. Therefore, it is critical to developing therapies with the ability to alleviate the effects of oxidative stress and intestinal barrier damage caused by sleep deprivation. 

Probiotics have been widely used to alleviate digestive systemic diseases. *Lactiplantibacillus plantarum* (*L. plantarum*), as one of the most widely used species, has shown multiple functions. Studies have demonstrated that *L. plantarum* exerts antioxidant functions through multiple pathways, such as the production of multiple antioxidant enzymes, chelation of metal ions, and alteration of gut microbiota [9,10,11]. *L. plantarum* DP189 delayed α-SYN accumulation in *N*-methyl-4-phenyl-1,2,3,6-tetrahydropyridine-induced Parkinson’s disease mice via regulating oxidative stress, immunity, and intestinal microbes [12]. *L. plantarum* KLDS1.0386 with antioxidant function ameliorates LPS-induced acute liver injury in mice through NF-κB and Nrf2 pathways [13]. It has been observed that *L. plantarum* AR113 alleviates DSS-induced colitis by modulating the TLR4-MyD88-NF-κB pathway and gut microbiota composition [14]. However, whether or not *L. plantarum* attenuates the intestinal damage induced by sleep deprivation remains unclear.

In this study, we used a sleep deprivation cage to establish a mouse model of sleep deprivation. Firstly, we selected different durations of SD to explore the effects of sleep deprivation duration on intestinal oxidative stress, inflammation, and the intestinal barrier in mice. Then, various indicators were measured to analyze the improvement of *L. plantarum* 124 on intestinal barrier damage and microbiota imbalance relative to SD. Finally, we explored the mechanism through which *L. plantarum* 124 alleviates the SD-associated impairment of intestinal barrier function.

## 2. Materials and Methods

### 2.1. Bacterial Strains

*L. plantarum* 124 was isolated from feces sampled from longevous people in the World’s Longevity Township and stored in Guangdong Microbial Species Preservation Center; its preservation number was GDMCC61123 [15]. The frozen strains were inoculated into 10 mL of Man Rogosa Sharpe (MRS, Guangdong Huankai Biotechnology Co., Ltd., Guangzhou, China) and incubated anaerobically at 37 °C for 24 h. Activated *L. plantarum* 124 was inoculated into 50 mL of MRS meat broth and incubated anaerobically at 37 °C for 24 h. Then, the samples were centrifuged at 5500× *g* for 10 min at 4 °C, and then the cells were retained, resuspended with normal saline, and prepared into a bacterial suspension of 10^10^ CFU/mL for subsequent studies.

### 2.2. Animals and Experiment Design 

All experiments were approved by the Management and Ethics Committee of Experimental Animals of the Institute of Microbiology, Guangdong Academy of Sciences. Two batches of specific pathogen-free (SPF) male C57/BL6J mice (25 ± 2.0 g, aged 6 weeks) were purchased from Guangdong Medical Experimental Animal Center (Guangzhou, Guangdong, China). The mice could eat and drink freely and were kept under conventional conditions for 1 week of acclimatization.

**Figure 1:**
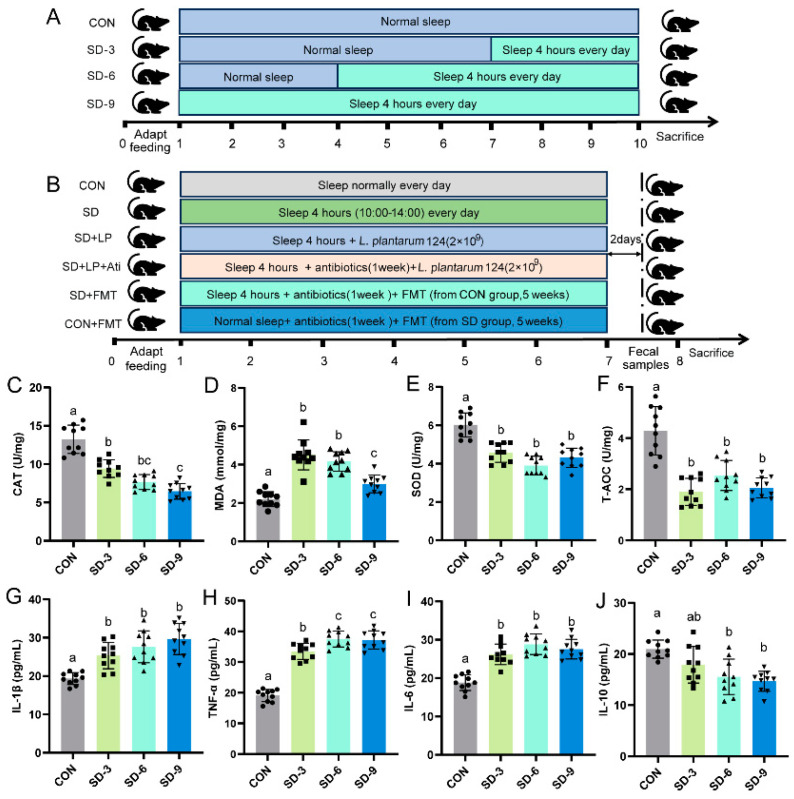
Effects of SD on colon oxidative stress and inflammation in mice. (**A**) Schematic representation of the first animal experiments; (**B**) schematic representation of the second batch of animal study; (**C**–**F**) antioxidant parameters: (**C**) CAT, (**D**) MDA, (**E**) SOD and (**F**) T-AOC; (**G**–**J**) cytokines: (**G**) IL-1β, (**H**) TNF-α, (**I**) IL-6, and (**J**) IL-10. Data are expressed as means ± SD (*n* = 10), and one-way ANOVA was used for comparison among multiple groups. Different letters represent significant differences in results (*p* < 0.05). CON: Control; SD-3: a duration of sleep deprivation of 3 weeks; SD-6: a duration of sleep deprivation of 6 weeks.

After the 7 days of acclimatization, the first batch of 48 mice was divided into four groups: the CON group consisted of mice placed in a sleep deprivation cage (Shanghai Xinruan Information Technology Co., Shanghai, China) and that had normal sleep conditions; the SD-3 group consisted of mice that usually slept for 6 weeks and then slept for 4 h (10:00–14:00) every day for 3 weeks in a sleep-deprived cage, while in the SD-6 group and the SD-9 group the mice had the same sleep deprivation as did those in the SD-3 group, but the duration was 6 weeks and 9 weeks, respectively (Figure 1A). After sleep deprivation, the mice were fasted, anesthetized with Shutai 50 (Virbac Group. Carros, France) the next day, and dissected. Serum, colon tissue, and intestinal contents were collected and divided into three parts for the subsequent measurement of indicators.

The second batch of 48 mice was divided into 6 groups and placed in sleep deprivation cages. Mice in the CON group were fed and slept freely every day; the SD group mouse slept for 4 h (10:00–14:00) a day for 6 weeks; the SD + LP group mice slept for 4 h a day, and then orally consumed *L. plantarum* 124 at a dosage of 2 × 10 ^9^ CFU/day for 5 weeks; the SD + LP + Ati group slept for 4 h per day and was pretreated with antibiotics for one week before the oral administration of *L. plantarum* 124 at a dosage of 2 × 10 ^9^ CFU/day for 5 weeks. In the SD + FMT group, the sleep-deprived mice were transplanted with fecal microbiota from normal sleep mice every other day for five weeks. The CON-FMT group mice were normal sleepers and transplanted fecal microbiota from the sleep-deprived mice every other day for five weeks. Two days after the last fecal microbiota transplantation, feces were collected from mice (Figure 1B). The mice were anesthetized with Shutai 50 (Virbac Group. Carros, France) and dissected. Serum, colon tissue, and intestinal contents were collected and divided into three parts for the subsequent determination of indicators.

### 2.3. Fecal Microbiota Transplantation (FMT)

Modified according to the previous literature [16,17], the feces of sleep-deprived mice were collected once every other day, all feces were pooled together, and then homogenized at 100 mg/mL and supplemented with saline (Solarbio Science & Technology Co., Ltd., Beijing, China). The homogenized solution was centrifuged at 500 rpm for 10 min, and the supernatant was the bacterial suspension. An amount of 100 μL/10 g of the bacterial suspension was transplanted into normal sleeping mice via oral gavage three times a week for 6 weeks. Before FMT, recipient mice were treated with 200 μL of an antibiotic mixture (ampicillin (Shanghai Macklin Biochemical Co., Ltd., Shanghai, China), 1 g/L; metronidazole (Shanghai Macklin Biochemical Co., Ltd., Shanghai, China), 1 g/L; neomycin (Shanghai Macklin Biochemical Co., Ltd., Shanghai, China), 1 g/L; vancomycin (Shanghai Macklin Biochemical Co., Ltd., Shanghai, China), 0.5 g/L) three times, one day apart. FMT was stopped two days before dissection. Fecal samples from the donor and recipient were collected in a sample stabilizer and cryopreserved for subsequent gut microbiota analysis.

### 2.4. Histological Analysis

Colonic morphology was observed using Hematoxylin–Eosin (H&E, Solarbio Science & Technology Co., Ltd., Beijing, China) and periodic acid–Schiff (PAS, Solarbio Science & Technology Co., Ltd., Beijing, China) staining [18]. After anesthesia, mice were dissected, and colon tissue was fixed with a 10% formaldehyde solution (Guangzhou yongjin Biotechnology Co., Ltd., Guangzhou, China). After 48 h, the colon tissue was dehydrated in an ethanol gradient, embedded in paraffin, cut into 5 μm thin sections, and finally stained with H&E or PAS. Intestinal morphology was observed and photographed using an inverted optical microscope. 

### 2.5. Biochemical Analyses

Mouse colonic tissue was collected, diluted with PBS (Solarbio Science & Technology Co., Ltd., Beijing, China) 10 times, and then placed in a low-temperature grinder for grinding. Under the conditions of 4 °C at 5000 rpm centrifugation for 10 min, the supernatant was transplanted on a new EP tube. After dilution, the levels of the protein content, superoxide dismutase (SOD), glutathione (GSH), catalase (CAT), malondialdehyde (MDA), and total antioxidant capacity (T-AOC) were determined using a BAC, SOD, GSH, CAT, MDA, and T-AOC determination kit (Nanjing Jiancheng Bio Co., Nanjing, China). 

### 2.6. Enzyme-Linked Immunosorbent Assay (ELISA) 

In accordance with the manufacturer’s instructions (Beijing winter song Boye Biotechnology Co., Ltd., Beijing, China), the content of inflammatory cytokines interleukin (IL)-1β, IL-6, IL-10, tumor necrosis factor-α (TNF-α), Occludin-1, and Claudin-1 were measured in intestinal samples. Colon samples were weighed at 50 mg, then 9 times the volume of physiological saline was added, grinded (at −30 °C, and at 60 Hz for 30 s, three times), then centrifuged at 13,000 rpm for 15 min at 4 °C, and the supernatant was transferred to a new EP tube for subsequent experiments. An amount of 50 μL of the supernatant was sucked into the sample well, and 100 μL of a horseradish-peroxidase-labeled detection antibody was added to each well. The samples were incubated at 37 °C for 60 min, and then the liquid was discarded and washed 5 times with washing solution. Briefly, 50 μL of substrate A (containing hydrogen peroxide) and B (containing TMB) was added and incubated at 37 °C in the dark for 15 min. Then, 50 μL of a termination solution was added and optical density was determined at a wavelength of 450 nm. In addition, serum levels of D-lactic acid (D-LA) and diamine oxidase (DAO) were measured using an ELISA kit (Beijing winter song Boye Biotechnology Co., Ltd., Beijing, China) in accordance with the above procedures.

### 2.7. Immunofluorescence Analysis

The mucin MUC2 in colon tissue was stained via immunohistochemistry. Briefly, paraffin sections of colon tissues were dewaxed with xylene and rehydrated through different concentrations of ethanol, then antigen repair was performed. After blocking with BSA, they were mixed with clonal rabbit anti-mouse primary antibody (MUC2, 1:1000; Beyotime) and incubated overnight at 4 °C. The cells were washed three times with PBS and incubated with goat anti-rabbit IgG H&L (Alexa Fluor^®^488) for 2 h at room temperature. After final washing as described above, tissue sections were sequentially stained with DAB and hematoxylin, dehydrated, and sealed with neutral resin. Intestinal morphology was observed and photographed using an inverted optical microscope.

### 2.8. Fecal DNA Extraction and 16S rRNA Gene Sequencing

Bacterial DNA was extracted from the fecal samples (0.2–0.3 g) using the E.Z.N.A. soil DNA kit (Omega Bio-tek, Norcross, GA, USA), and the V3–V4 variable regions of bacterial 16S rRNA genes were amplified using an ABI GeneAmp 9700 PCR thermocycler (ABI, Waltham, MA, USA). PCR products were purified using the AxyPrep DNA gel Extraction Kit (AxyPrep Biosciences, Union City, CA, USA). The purified products were quantified with a NanoDrop 2000 spectrophotometer (Thermo Fisher Scientific, Waltham, MA, USA) and a quantum fluorometer (Promega, Madison, WI, USA). Libraries were constructed, then sequenced using the Illumina MiSeq PE300 platform (Illumina, San Diego, CA, USA).

### 2.9. Gut Microbiota Analysis

All sequences were merged, quality-filtered, denoised, and flattened, and the ASV table was obtained in accordance with the Silva 16S rRNA gene database (v138) [19]. Based on the ASV table, the samples were analyzed through the online platform Majorbio Cloud Platform (www.majorbio.com, accessed on 28 September 2022). The analysis content included Alpha diversity analysis and Beta diversity analysis. Multivariate principal component analysis (PCA) and non-metric multidimensional scaling (NMDS) analysis were used to investigate the similarities or differences in community composition and to identify the potential principal components that affected the differences in community composition. A Circos diagram was used to visualize the distribution of dominant gut microbiota species at the genus level. A community heat map was used to visually visualize the differences in gut microbiota abundance at thew genus level. Finally, accordingly, the Bar map was used to explore the differences in microbial community abundance between each sample group at the phylum level and genus level.

### 2.10. Statistical Analysis

Based on Majorbio Cloud Platform (www.majorbio.com), the non-parametric Kruskal–Wallis test and Mann–Whitney test or Dunn test were used to analyze the data on gut microbiota composition. The data statistics involved using GraphPad Prism (GraphPad Software v8.0.2). All data were expressed as means ± SD. Significant differences among groups were determined via a one-way analysis of variance (ANOVA) with Duncan’s range tests. The statistical significance was set to *p* < 0.05.

## 3. Results

### 3.1. Sleep Deprivation Is Associated with Oxidative Stress and Inflammation in the Gut

Studies have reported that intestinal inflammation and sleep deprivation have a high degree of clinical comorbidity [20]. Alexandra Vaccaro et al. revealed that sleep deprivation causes early death through the accumulation of oxidative stress in the gut [6]. To investigate the effects of SD on intestinal oxidative stress and the inflammatory response, we first constructed a sleep-deprived mouse model through a sleep deprivation cage and examined the changes in antioxidant enzymes and inflammatory factors in colon tissues. Compared with the CON group, the levels of antioxidant enzymes (SOD, CAT, and T-AOC) in the SD group were significantly decreased, while the level of MDA was significantly increased (*p* < 0.05, Figure 1C–F). The changes in colonic antioxidant enzymes correlated with the sleep deprivation duration. In addition, measurements of intestinal inflammatory factors showed that proinflammatory factors (IL-1β, IL-6, TNF-α) were significantly increased, and anti-inflammatory factors (IL-10) were significantly decreased in the sleep-deprived group compared with those in the CON group (*p* < 0.05, Figure 1G–J). These results indicated that insufficient sleep is associated with intestinal oxidative stress and inflammation.

### 3.2. Effects of Sleep Deprivation on the Intestinal Barrier

**Figure 2:**
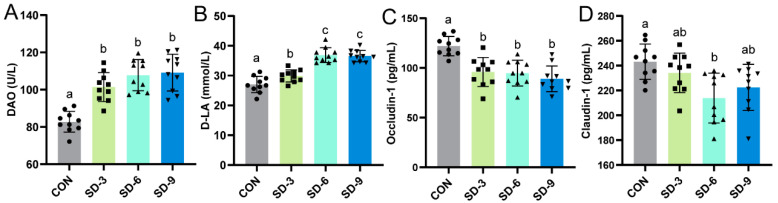
Sleep deprivation impairing colon barrier integrity in mice. (**A**) DAO; (**B**) D-LA; (**C**,**D**) tight junction protein: (**C**) Occludin-1 and (**D**) Claudin-1. Data are expressed as means ± SD (*n* = 10), and one-way ANOVA was used for comparison among multiple groups. Different letters represent significant differences in results (*p* < 0.05). CON: control; SD-3: a duration of sleep deprivation of 3 weeks; SD-6: a duration of sleep deprivation of 6 weeks.

Next, we assessed whether or not SD affected intestinal barrier permeability. DAO and D-LA serum levels are biomarkers of intestinal barrier permeability and intestinal wall integrity [21,22]. The results showed that compared with those in the CON group, the serum levels of DAO and D-LA in the SD group were significantly increased, indicating that intestinal permeability was increased in the sleep-deprived mice (*p* < 0.05, Figure 2A,B). In addition, the results of the intestinal tight junction protein measurement showed that the contents of Claudin-1 and Occludin-1 in the SD group decreased compared to those in the CON group, especially in the SD-6 and SD-9 group which showed a significant decrease (*p* < 0.05, Figure 2C,D). These results indicate that the integrity of the intestinal barrier decreases with the prolongation of sleep deprivation. In summary, we tentatively judged that SD was associated with increased intestinal barrier permeability.

### 3.3. L. plantarum 124 Alleviated SD-Associated Inflammation and Oxidative Stress in the Colon

**Figure 3:**
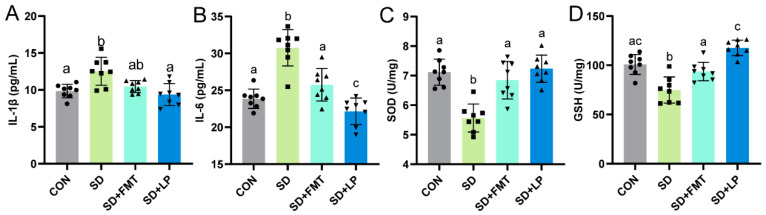
Supplementation of *L. plantarum* 124 alleviating SD-associated changes in colonic inflammatory factors and oxidative stress. (**A**,**B**) cytokines antioxidant parameters: (**A**) IL-1β and (**B**) IL-6; (**C**,**D**) antioxidant parameters: (**C**) SOD and (**D**) GSH. Data are expressed as means ± SD (*n* = 8), and one-way ANOVA was used for comparison among multiple groups. Different letters represent significant differences in results (*p* < 0.05). CON: control; SD: sleep deprivation; SD + FMT: sleep-deprived mice transplanted with fecal microbiota from normal-sleeping mice; SD + LP: sleep-deprived mice supplemented with *L. plantarum* 124.

To investigate whether or not supplementation with *L. plantarum* 124 and intestinal microbiota transplantation affect SD-associated colonic injury, we examined changes in intestinal inflammation and oxidative stress levels in sleep-deprived mice after *L. plantarum* 124 supplementation or transplanting gut normal microbiota from normal-sleeping mice, respectively. Compared with the SD group, *L. plantarum* 124-supplemented mice had a significant decrease in colonic proinflammatory factor IL-1β and a significant increase in anti-inflammatory factor IL-6 (*p* < 0.05, Figure 3A,B). In terms of intestinal oxidative stress, *L. plantarum* 124 supplementation reversed the SD-associated decrease in SOD and GSH in the colon of mice (*p* < 0.05, Figure 3C,D). These data suggested that *L. plantarum* 124 can enhance the antioxidant effect of colon tissue, maintain antioxidant balance in SD mice, and partially reversed the intestinal oxidative stress and inflammation response associated with SD. In addition, intestinal oxidative stress and inflammation were significantly alleviated in the SD + FMT group (*p* < 0.05, Figure 3A–D). The above results suggest that the gut microbiota has a specific function in alleviating intestinal oxidative stress and inflammation associated with SD.

### 3.4. Effect of L. plantarum 124 on the SD-Associated Intestinal Barrier

**Figure 4:**
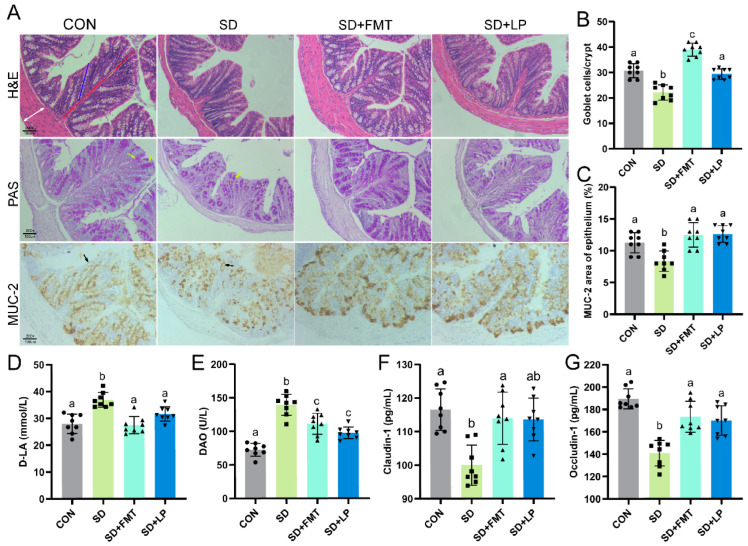
*L. plantarum* 124 alleviating SD-associated intestinal barrier damage. (**A**) Histopathological changes in the colon, mucosal thickness (red line), crypt depth (blue line), colon wall (white arrow), goblet cell (yellow arrow), and MUC2 (black arrow), magnification ×200; (**B**) the number of goblet cells in the colon; (**C**) MUC2 quantified via immunohistochemical staining in the colon of mice. (**D**,**E**) indicators of intestinal permeability: (**D**) D-LA and (**E**) DAO; (**F**,**G**) tight junction protein: (**F**) Occludin-1; (**G**) Claudin-1. Data are expressed as means ± SD (*n* = 8), and one-way ANOVA was used for comparison among multiple groups. Different letters represent significant differences in results (*p* < 0.05). CON: control; SD: sleep deprivation; SD + FMT: sleep-deprived mice transplanted with fecal microbiota from normal-sleeping mice; SD + LP: sleep-deprived mice supplemented with *L. plantarum* 124.

The results showed that sleep deprivation is associated with increased intestinal barrier permeability. Therefore, we further analyzed the effect of *L. plantarum* 124 intervention on the intestinal barrier. Compared with the SD group, H&E staining showed that the mucosal thickness (red line), crypt depth (blue line), and muscle layer thickness (white arrow) in the SD + LP group were significantly improved (*p* < 0.05, Figure 4A). Intestinal permeability was measured by evaluating the leakage of DAO and D-LA. Results revealed a significant increase in DAO and D-LA levels in the serum of mice in the SD group. However, with the administration of *L. plantarum* 124, these levels significantly decreased (*p* < 0.05, Figure 4D,E). Interestingly, normal sleeping mouse fecal recipient SD mice also exhibited a similar phenomenon. In addition, we evaluated the effects of *L. plantarum* 124 supplementation or transplanted normal-sleeping mouse intestinal microbiota on the intestinal integrity of SD mice by measuring the content of tight junction proteins (Occludin-1, and Claudin-1) in colon tissue. Compared with that in the CON group, tight junction protein content in the SD group was significantly decreased, whereas that in the SD + LP group and SD + FMT group were significantly up-regulated (*p* < 0.05, Figure 4F,G). Moreover, the effect of the transplantation of normal microflora on SD-associated injury was superior to that of *L. plantarum* 124 supplementation. Of note, goblet cells and MUC2 protein are essential for maintaining the integrity of the intestinal barrier. Goblet cells in the colon were visualized with PAS staining (Figure 4A,B). The results showed that the number of goblet cells in mice fed *L. plantarum* 124 significantly increased than that in the SD group. In addition, the immunohistochemical staining of the colon tissues of mice in each group showed that the expression of MUC2 protein was decreased in sleep-deprived mice. At the same time, it was increased with the *L. plantarum* 124 supplement (*p* < 0.05, Figure 4A,C). Taken together, these results suggested that *L. plantarum* 124 intervention or the transplantation of gut microbiota from normal sleeping mice was effective in repairing the intestinal barrier disruption associated with sleep deprivation. However, whether or not *L. plantarum* 124 was directly related to gut microbiota was still unknown.

### 3.5. L. plantarum 124 Regulated the Homeostatic Balance of the Gut Microbiota

**Figure 5:**
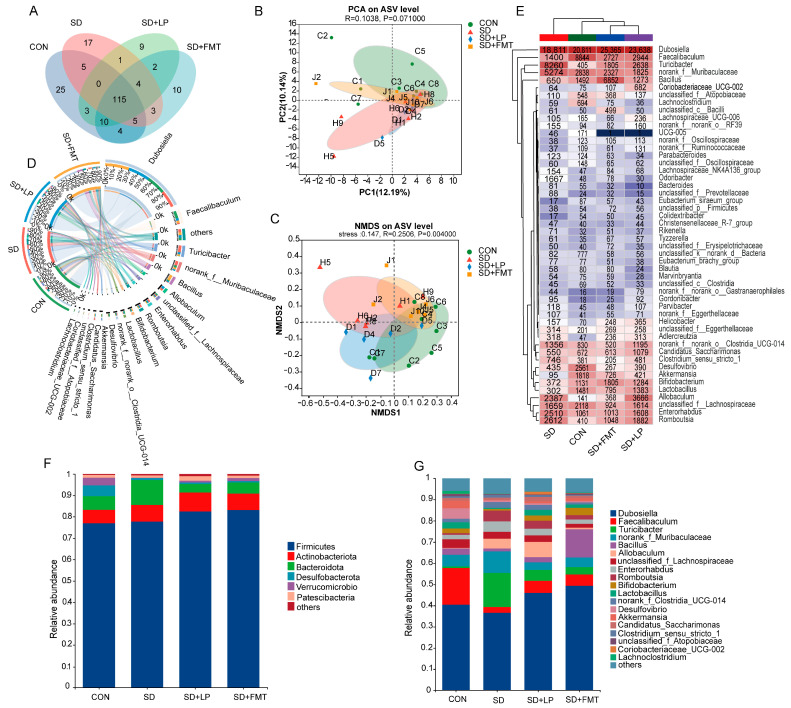
*L. plantarum 124* regulating gut microbiota composition in sleep-deprived mice. (**A**) Venn diagram showing the genus levels in gut microbiota among groups. (**B**) PCA analysis. (**C**) NMDS analysis. (**D**) Circos diagram of the gut microbiota at the genus level. (**E**) Genus-level abundance heatmap of gut microbiota. (**F**,**G**) Relative abundance at phylum and genus levels. CON: control; SD: sleep deprivation; SD + FMT: sleep-deprived mice transplanted with fecal microbiota from normal-sleeping mice; SD + LP: sleep-deprived mice supplemented with *L. plantarum* 124.

Previous studies have confirmed the association between sleep deprivation and the gut microbiome. This study found that transplanting the gut microbiota of normal-sleeping mice alleviated SD-associated changes in colonic phenotypes. This provides evidence that gut microbes may have a function in the treatment of sleep deprivation-related injuries. Therefore, we identified the effect of *L. plantarum* 124 intervention on microbiota composition via 16S rRNA gene amplicon pyrosequencing. The chimeric sequences were identified and removed, resulting in a total of 10,966 ASVs. An average of 3527, 2463, 2071 and 2905 ASVs were obtained in the CON, SD, SD + LP and SD + FMT groups, respectively. A total of 614 genera were identified, and a Venn diagram showed that on average 167, 150, 144, and 153 genera were identified in the CON, SD, SD + LP, and SD + FMT groups, respectively (Figure 5A). Moreover, principal component analysis (PCA, Figure 5B) and non-metric multidimensional scaling (NMDS, Figure 5C) showed the obvious clustering of gut microbiota composition in the four groups. The changes in the intervention group tended toward the CON group, indicating that the supplementation of *L. plantarum* 124 and FMT changed the structure of SD-associated gut microbiota in mice. The Circos chart showed that the most dominant genera in all experimental groups were *Dubosiella*, *Faecalibaculum*, *Turicibacter*, *norank f Muribaculaceae*, *Bacillus*, and unclassified *f Lachnospiraceae* (Figure 5D). At the genus level, a cluster analysis of the top 50 species showed that the SD + LP group and SD + FMT group were clustered into the same group and then with the CON group (Figure 5E).

The phylum- and genus-level analysis showed that sleep deprivation significantly increased the relative abundance of *Bacteroidetes* and decreased the relative abundance of *Firmicutes*, and that the supplementation of *L. plantarum* 124 or FMT showed improvement (Figure 5F). At the genus level, compared to the levels of those in the CON group, a significant decrease in *Dubosiella, Faecalibaculum*, *Bacillus*, *Bifidobacterium*, *Akkermansia*, *Intestinimonas*, *Lactobacillus*, *Actinobacteriota*, *Colidextribacter*, and *Oscillospiraceae* levels was observed in the SD group. In the SD + LP and SD + FMT groups, compared with those in the SD group, the horizontal *Dubosiella*, *Faecalibaculum*, *Bifidobacterium*, *Colidextribacter*, *Akkermansia*, and *Lactobacillus* levels were significantly enriched (Figure 5G). In addition, compared to that in the CON group, the SD treatment resulted in a higher abundance of *Erysipelotrichaceae*, *Bacteroides*, *Odoribacter*, *Romboutsia*, *Streptococcus*, *Clostridium_sensu_stricto_1 Erysipelatoclostridium*, *Prevotellaceae*, and *Enterorhabdus*. In contrast, *L. plantarum* 124 administration or FMT reduced the abundance of *Erysipelotrichaceae, Odoribacter*, *Romboutsia*, *Streptococcus*, and *Erysipelatoclostridium*. These results suggested that *L. plantarum* 124 supplementation mitigated SD-associated colonic microbiota disorder in mice.

### 3.6. Gut Microbiota Mediated the Effects of L. plantarum 124 on Alleviating Sleep-Deprivation-Related Intestinal Damage 

**Figure 6:**
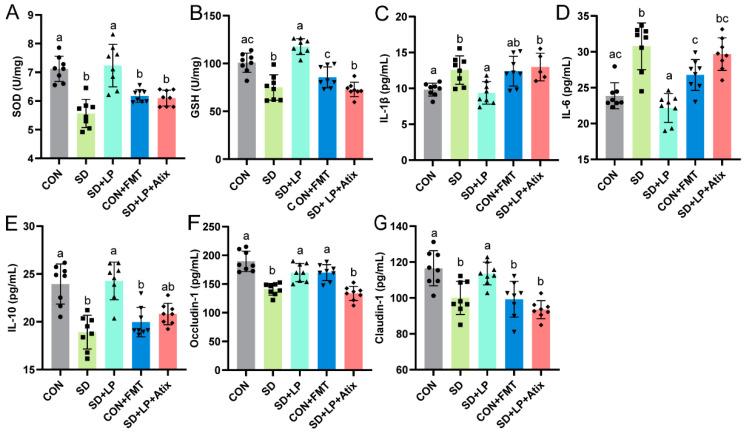
Gut microbiota mediating the function of *Lactobacillus plantarum* 124 in alleviating SD-related intestinal damage. (**A**,**B**) Antioxidant parameters: (**A**) SOD and (**B**) GSH; (**C**,**E**) cytokines: (**C**) IL-1β, (**D**) IL-6 and (**E**) IL-10; (**F**,**G**) tight junction protein: (**F**) Occludin-1 and (**G**) Claudin-1. Data are expressed as means ± SD (*n* = 8), and one-way ANOVA was used for comparison among multiple groups. Different letters represent significant differences in results (*p* < 0.05). CON: control; SD: sleep deprivation; SD + LP: sleep-deprived mice were supplemented with *L. plantarum* 124; CON + FMT: normal-sleeping mice transplanted with fecal microbiota from sleep-deprived mice; SD + LP + Atix: sleep-deprived mice pretreated with antibiotics before supplementation with *L. plantarum* 124.

In the previous study, *L. plantarum* 124 reversed the colonic oxidative stress, inflammation (Figure 3A–D), intestinal barrier damage (Figure 4), and gut microbiota disorder caused by SD to a certain extent. We used antibiotic treatment and fecal microbiota transplantation pretreatment on sleep-deprived mice to elucidate further the causal relationship between *L. plantarum* 124, gut microbiota, and intestinal injury. As shown, after transplanting the gut microbiota of sleep-deprived mice, intestinal oxidative stress levels, inflammatory factors, and intestinal permeability indexes of the CON + FMT group tended to reflect those of sleep-deprived mice (Figure 6). The result suggested that gut microbiota mediated the gut damage caused by sleep deprivation. Compared with the SD group, *L. plantarum* 124 supplementation alleviated intestinal damage. Interestingly, pretreatment with a quadruple antibiotic eliminated the repair effects of *L. plantarum* 124 on intestinal barrier damage in sleep-deprived mice. Therefore, *L. plantarum* 124 ameliorated intestinal injury in sleep-deprived mice, possibly by regulating gut microbiota.

## 4. Discussion

Sleep is a necessary physiological process in maintaining the homeostasis of the endocrine and immune systems [23]. Sleep deprivation increases the risk of diseases, such as cardiovascular and nervous system diseases, which may also induce chronic metabolic disorders [24,25,26]. Clinical evidence shows that sleep deprivation and intestinal disease are highly comorbid [20]. Here, we selected to examine sleep deprivation mouse models of different duration times to explore the effects of sleep deprivation on the intestinal injury. We measured the changes in oxidative stress and inflammatory factors in colon tissues. Sleep deprivation has been reported to cause an accumulation of ROS in the gut. The results of the present study showed that compared with those in the CON group, the activities of SOD, CAT and T-AOC in the colon of the SD group were significantly decreased (*p* < 0.05), and MDA content significantly increased (*p* < 0.05). In organisms, GSH-Px, SOD, and CAT have the function of scavenging harmful ROS and maintaining oxidative stress balance [27]. The accumulation of ROS results in the production of MDA. Studies have proven that excessive MDA induces NO production, leading to the infiltration of many inflammatory cells in the colon tissues [28]. In addition, clinical studies have shown that sleep deprivation promotes the production of inflammatory cytokines in peripheral blood, indicating that sleep deprivation leads to systemic inflammation [29]. 

In our study, the colonic proinflammatory factors (IL-1β, IL-6 and TNF-α) were significantly increased, while the anti-inflammatory factor (IL-10) was significantly decreased in the SD-3, SD-6 and SD-9 groups, which was consistent with the results of previous studies [27]. In conclusion, these results suggest that sleep deprivation is associated with oxidative stress and inflammation in the colon, and the exact mechanisms need to be further studied.

*Lactiplantibacillus plantarum* has anti-inflammatory properties and antioxidant capacity and is commonly used to prevent intestinal and metabolic diseases [13]. In this study, we evaluated the mitigating effects of *L. plantarum* 124 on intestinal oxidative stress, inflammation, and barrier damage associated with sleep deprivation and analyzed the mechanism of action. Our data showed a significant reduction in antioxidant enzyme content in the SD group, whereas the opposite trend was observed in the SD + LP group, indicating that *L. plantarum* 124 reverses the oxidative stress imbalance associated with sleep deprivation. Consistent with the above results, supplementation with *L. plantarum* 124 alleviated the upregulation of proinflammatory cytokines (IL-1β and IL-6) associated with SD. 

The intestinal barrier consists of the mucous layer, symbiotic bacteria, epithelial cells, and immune cells, which protect the host from toxins and microorganisms in the intestinal lumen. Intestinal permeability is a direct indicator of the integrity of the intestinal barrier [30]. DAO and D-LA are usually only present in the intestinal lumen and can enter the serum when the intestinal barrier is damaged. Our results showed that supplementation with *L. plantarum* 124 reduced the contents of DAO and D-LA in the serum of SD-treated mice. Tight junction protein has the function of connecting epithelial cells and controlling paracellular permeability. The study showed that SD decreased the content of tight-junction-related proteins Claudin-1, and Occludin-1, which could be recovered after treatment with *L. plantarum* 124. The histological morphology of the colon was observed via H&E staining, and Figure 4A shows that the colon wall was thinner and the crypt depth was shallower in the SD group. Goblet cells have the function of secreting mucus and protecting the integrity of the intestinal barrier. Colonic goblet cells can be observed explicitly via PAS staining. In the present study, goblet cells were counted after PAS staining. The results showed that the SD group had significantly fewer goblet cells than did the CON and SD + LP groups. MUC2 mucin, as a key component of late mucus, plays a crucial role in protecting the intestine and maintaining intestinal homeostasis. Pathogenic bacteria can degrade MUC2 mucins through different mechanisms to disrupt the intestinal barrier. For example, enterotoxigenic *Escherichia coli* induces intestinal mucosal barrier breakdown by secreting mucin-degrading metalloprotease YghJ [31,32]. Similarly, the intestinal barrier in mice was enhanced via intervention to promote MUC2 mucin transcription in different areas of the gut. In our study, sleep deprivation resulted in a significant decrease in MUC2, whereas it increased dramatically after the intervention with *L. plantarum* 124 (*p* < 0.05, Figure 4). Therefore, our results suggested that *L. plantarum* 124 intervention was effective in repairing sleep-deprivation-associated intestinal barrier damage. Our previous study found that L-ascorbate and mesaconic acid were significantly enriched in the serum of *L. plantarum* 124-treated animals. Analysis of the metabolites and genome-wide data of *L. plantarum* 124 revealed its ability to synthesize L-ascorbic acid and mescononic acid [15]. L-ascorbate and mesaconic acid are known to have powerful antioxidant effects [33]. We speculate that L-ascorbate and mesaconic acid are the material basis for the function of *L. plantarum* 124.

Interestingly, transplanting the gut microbiota from normal-sleeping mice partially reversed SD-associated intestinal oxidative stress, inflammation, and intestinal barrier damage. This finding reveals that the gut microbiota is essential in the relationship between sleep and gut health. Multiple studies have confirmed that sleep deprivation causes changes in the gut microbiome. However, there was no significant difference of gut microbiota abundance in our study. An analysis of gut microbiota composition showed that *Intestinimonas, Dubosiella, Faecalibaculum, Bifidobacterium, Colidextribacter, Akkermansia, Oscillospiraceae*, and *Lactobacillus* were significantly enriched in SD + LP and SD + FMT groups. Studies have shown that *Faecalibaculum* is anti-inflammatory, protecting the digestive system from intestinal pathogens [34]. Lukas F Mager et al. found a significant decrease in the abundance of *Colidextribacter* in early and middle colorectal cancer [35]. In addition, the *Intestinimonas* bacteria, a vital butyrate producer, has anti-inflammatory and antioxidant properties [13,36]. *Bifidobacterium*, *Akkermansia*, and *Lactobacillus*, as the main source of probiotics, protect the body’s intestinal barrier. In addition, in this study, supplementation with *L. plantarum* 124 or the transplantation of normal sleeping gut microbiota decreased the relative abundance of *Erysipelotrichaceae*, *Bacteroides*, *Odoribacter*, *Romboutsia*, *Streptococcus*, *Erysipelatoclostridium*, *Prevotellaceae* and *Enterorhabdus* genus caused by SD. As for the mucosal microbiota of intestinal polyps, Marta Mangifesta et al. revealed that the relative abundance of *Romboutsia* was closely related to the occurrence of intestinal adenomatous polyps [37]. Jian Sun et al. found that *Erysipelaceae* and *Bacteroidetes* were the key strains of ulcerative colitis (UC) [38]. Su et al. demonstrated that intestinal types rich in *Prevotellaceae* might be positively associated with a higher risk of diarrhea-dominated irritable bowel syndrome [39]. In addition, multiple studies have shown that the relative abundance of *Prevotellaceae* and *Odoribacter* correlates with mild diarrhea and gut microbiota dysbiosis following berberine treatment. In summary, the results suggest that *L. plantarum* 124 and FMT intervention have the potential to reshape the changes in the relative abundance of gut microbiota associated with SD.

Accumulating evidence suggests that probiotic supplementation can be used to treat intestinal barrier damage via alleviating imbalances in the gut microbiome. For example, *L. plantarum* AR113 ameliorates DSS-induced colitis by regulating the TLR4-MyD88-NF-κB pathway and gut microbiota composition [14]. Yongjun Xia et al. revealed that *L. plantarum* 1201 alleviates inflammatory bowel disease through partially restoring the gut microbiota, upregulating serum α-T and D-mannose content, protecting the intestinal barrier, and up-regulating immune function [40]. Our results reveal that the indexes of intestinal oxidative stress, inflammation, and intestinal damage in the CON + FMT group tended to reflect those in the SD group, consistent with Wang Zhong’s results [41], suggesting that sleep deprivation caused intestinal damage through the gut microbiota. In addition, supplementation with *L. plantarum* 124 did not repair SD-induced intestinal damage after antibiotics depleted the gut microbiota. Therefore, we hypothesized that *L. plantarum* 124 ameliorates intestinal damage through regulating the gut microbiota. In summary, the results suggest that the gut microbiota mediated *L. plantarum* 124 in alleviating SD-related intestinal barrier damage, but the effect and mechanism of action need to be further verified.

This is the first study to examine the effects of different durations of sleep deprivation on the gut microbiota, gut morphology, and physicochemical parameters. In addition, this study has strengths of visually demonstrating the role of *L. plantarum* 124 in attenuating sleep-related intestinal injury through gut microbiota using fecal microbiota transplantation and antibiotic pretreatment. However, this study has limitations. Data on the effects of *L. plantarum* 124 on gut microbiota metabolites were missing in the study. The specific molecular mechanisms and pathways underlying the action of *L. plantarum* 124 require further elucidation. We tentatively suggest that *L. plantarum* 124 plays a role in alleviating sleep-related intestinal damage by regulating the composition of the host gut microbiota and its own production of L-ascorbic acid and mescononic acid. In the future, the specific molecular mechanisms of *L. plantarum* 124 reducing sleep-deprivation-related intestinal injury will be analyzed based on metabolomics, metagenomics, and transcriptome data.

## 5. Conclusions

In summary, this study verified that SD is associated with intestinal oxidative stress, inflammation, and intestinal barrier damage in mice. Supplementation with *L. plantarum* 124 can improve oxidative stress levels, regulate the content of intestinal inflammatory factors, reduce intestinal barrier damage, and alleviate the imbalance of the intestinal microbiome in SD mice. The results of this study provide a new idea for probiotics to alleviate SD-related intestinal damage

## Data Availability

16S amplicon data from animal feces of C57/BL6J mice https://dataview.ncbi.nlm.nih.gov/object/PRJNA997797?reviewer (accessed on 24 July 2023). Submission ID: SUB13702060, BioProject ID: PRJNA997797.
